# Copper catalysis: a constantly evolving field

**DOI:** 10.3762/bjoc.21.109

**Published:** 2025-07-17

**Authors:** Elena Fernández, Jaesook Yun

**Affiliations:** 1 Departament de Química Física i Inorgànica, University Rovira i Virgili, Tarragona, Spain; 2 Department of Chemistry, Sungkyunkwan University, Suwon 16419, Republic of Korea

**Keywords:** copper, copper catalysis

Copper catalysis continues to thrive as one of the most dynamic and versatile areas of contemporary chemical research. Once viewed primarily as a cost-effective alternative to noble metals, copper has emerged as a powerful and versatile catalyst, capable of mediating a wide array of chemical transformations through both two-electron and single-electron pathways. This duality has enabled access to previously elusive molecular transformations, positioning copper at the center of modern synthetic strategy.

This thematic issue captures the dynamic nature of the field, featuring five insightful Review articles and five original research papers (three Full Research Papers and two Letters) contributed by scientists from Asia and Europe. The breadth of topics and the geographical diversity of the authors reflect the global interest in copper catalysis today.

The Review article by Yang and Fang focuses on copper-catalyzed yne–allylic substitution reactions [1]. In particular, the authors elaborate the concept and recent developments in this field, which allows access to enantioenriched chiral enynes. Interestingly, the article also illustrates the effects of the copper salt and the ligand employed, as well as the influence of the substrate substitution pattern on the regioselectivity and stereoselectivity of the reactions. A Review article by Papis and co-workers discusses various copper(II) triflate-catalyzed multicomponent reaction types [2]. Therein, the synthesis of cyclic and acyclic compounds, as well as three-component and four-component reactions, are separately debated. In addition, mechanistic insights into the reaction of heteropolycyclic systems, cycloadditions, and aza-Diels–Alder reactions are provided. This is important because multicomponent, one-pot reactions have gained interest as potentially more economic, efficient, and straightforward reactions. Complementarily, the Review article by Jang and Kim provides a deep understanding of recent advances in the combination of electrochemistry and copper catalysis for various organic transformations [3]. Their contribution elaborates various C–H functionalizations, olefin additions, decarboxylative functionalizations, and Chan–Lam coupling reactions. In doing so, the authors point out the combination of transition-metal catalysis and electrochemistry as an efficient, sustainable method for the oftentimes challenging formation of C–C and C–heteroatom bonds in complex molecules. Another Review by Son and co-workers illustrates recent advancements in the use of dioxazolones in synthetic transformations with copper salts [4]. The authors remark that these catalytic systems, which employ dioxazolones as electrophilic amide sources, were applied for the preparation of diverse amidated products. In their contribution, Son et al. focus on transformations via the formation of copper nitrenoids, particularly amidations via oxidative insertion to N–O bonds and reductive elimination, and a small number of other reactions. The final Review by Cho, Lee, and co-workers is useful for the scientific community in that it elegantly reviews recent advances in allylation reactions of copper-catalyzed asymmetric allylic substitution reactions of chiral secondary alkylcopper species [5]. In summary, the contribution includes stereospecific transmetalations of organolithium and -boron compounds, copper hydride catalysis, and enantiotopic group-selective allylations of 1,1-diborylalkanes as core strategies. At the same time, the authors provide detailed mechanistic insights into the stereocontrol and provide a perspective on currently unresolved challenges in the field.

Concerning Full Research Papers, Burley, Watson, and co-workers present a new synthesis of germyl triazoles from germyl alkynes through a copper-catalyzed azide–alkyne cycloaddition (CuAAC) reaction [6]. The resulting Ge-substituted triazoles could be further diversified. For example, through chemoselective transition-metal-catalyzed cross-coupling reactions of bifunctional boryl- and germyl-containing compounds. On the other hand, a Full Research Paper presented by Lee and co-workers introduces a highly regioselective formal hydrocyanation method of allenes [7]. The strategy is based on a copper-catalyzed hydroalumination of allenes with diisobutylaluminum hydride, which is followed by the allylation with *p*-toluenesulfonyl cyanide in a regio- and stereoselective manner. They propose a new way to access accessing acyclic β,γ-unsaturated nitriles with α-all-carbon quaternary centers. In the process, they managed to achieve a yield of up to 99%, with excellent regio- and *E*-selectivities. The method could be used for functional-group transformations of amines, amides, and lactams, as well as for gram-scale syntheses, demonstrating synthetic usefulness and versatility. A complementary Full Research Paper by Martina and co-workers demonstrates that the efficient and green direct C–H amination of benzoxazoles can be catalyzed by copper chloride salts in acetonitrile in the absence of any acidic, basic, or oxidizing additives [8]. Both CuCl and CuCl_2_ were found to be extremely efficient in promoting the reactions, which harness their Lewis-acidic and weakly oxidizing properties, respectively. In addition, microwave irradiation increases the reaction rate considerably. Furthermore, the use of a solid Cu(I) catalyst immobilized on an aminated silica support allows for a heterogeneous and cost-effective process, featuring straightforward workup and minimized free copper on solution. Due to this, the catalyst could be regenerated and reused in up to eight cycles. Upon optimization, this practical and versatile method could be used for the synthesis of several benzoxazole derivatives.

A Letter was contributed by D’Andrea and Jademyr, who focus on the preparation of phenethylamines and phenylisopropylamines via reduction of substituted β-nitrostyrenes using a system of sodium borohydride and copper(II) chloride [9]. The transformations are performed in one pot and proceed under mild conditions. The β-nitrostyrene products could be isolated quickly and in good yield, without the need for time-consuming purification steps. At the same time, no cumbersome precautions, such as an inert atmosphere, are necessary. Another fantastic Letter was contributed by Fañanás-Mastral and co-workers, in which a rare copper-catalyzed dimerization process of 4,4-dichloro-2-butenoic acid derivatives and bis(pinacolato)diboron is described [10]. The transformations feature excellent chemo-, regio-, and diastereoselectivities, and the resulting products are highly functionalized. Due to this, they were considered as versatile building blocks for the stereoselective synthesis of chlorocyclopropanes.

As guest editors, we are deeply grateful to all contributors for sharing their high-quality work and valuable perspectives. We also thank the referees for their thoughtful evaluations, which helped maintain the scientific rigor of this collection. We hope this collection inspires further exploration and innovation in this rapidly advancing field.

Elena Fernández and Jaesook Yun

Tarragona and Suwon, June 2025

## Data Availability

Data sharing is not applicable as no new data was generated or analyzed in this study.
